# Supplementary figures and images for: Viscoelastic properties of wheat gluten in a molecular dynamics study
Source: PLoS Comput Biol. 2021 Mar 24;17(3):e1008840. doi: 10.1371/journal.pcbi.1008840 (PMC8021197; doi:10.1371/journal.pcbi.1008840)

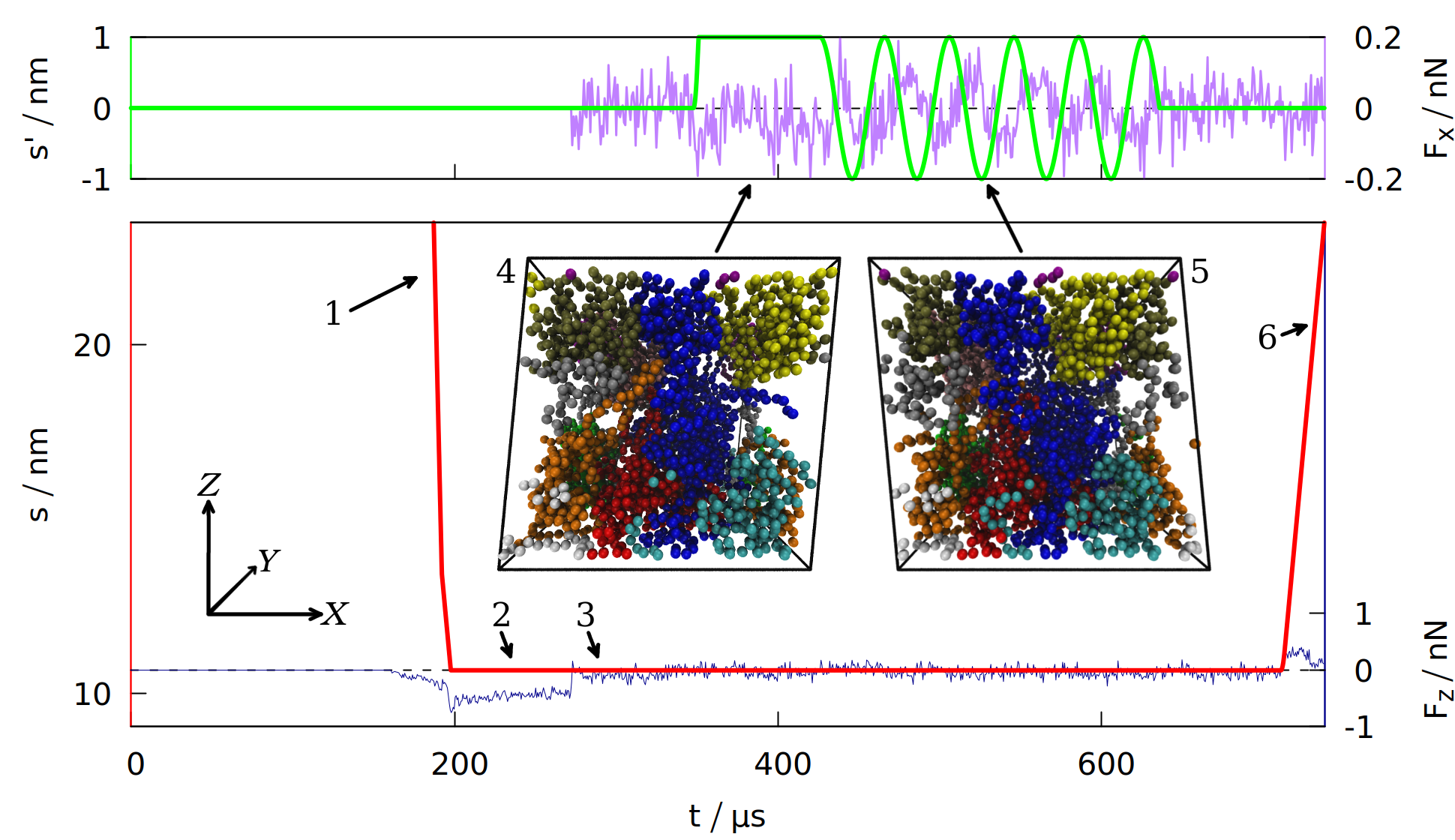

Supplement: S1 Fig — The purple curve (top panel) shows the X component of the force exerted on the proteins by the walls (summed over all the residues and averaged over 100 ns time interval), the blue curve (bottom panel) shows the Z component of that force. The data is for gluten (defined in Section 1 of the S1 Text), with the density of 3.5 nm−3. The period of oscillations is 40 ms. The numbers with arrows correspond to the stages of the simulation shown on Fig 1 in the main article. Snapshots 4 and 5 show the system during shearing oscillations (each bead represents one amino acid, the protein chains are shown in different colors). The coordinate system (top left) is the same as in Fig 1. (TIF) [file pcbi.1008840.s001.tif]

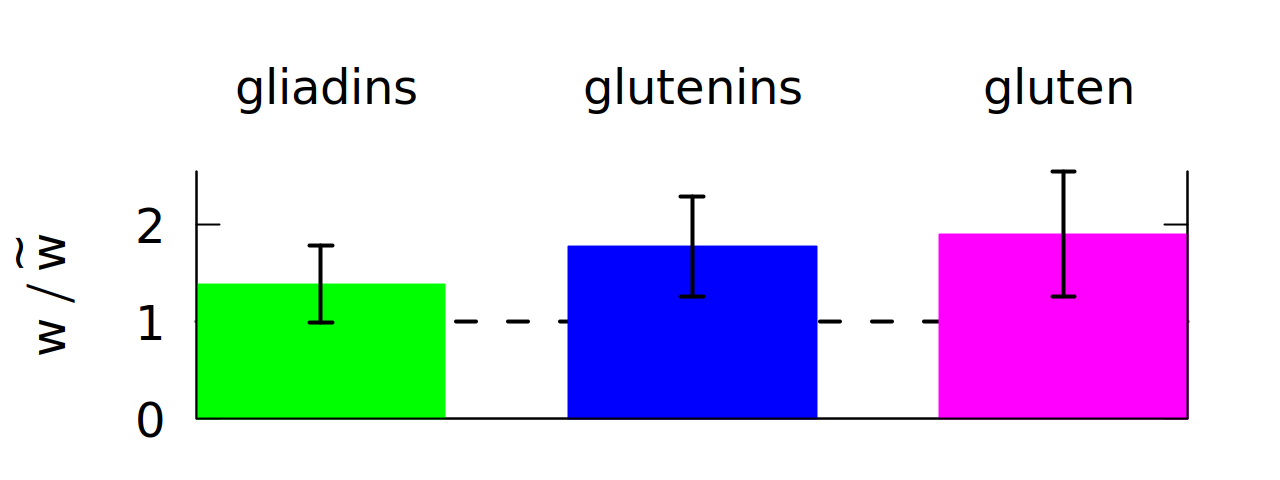

Supplement: S2 Fig — Period of oscillations is 70 μs (lower periods did not result in such significant changes in w). The ratio of 1 is marked by a broken line. Each color corresponds to a different system, as displayed at the top, and defined in Section 1 of the S1 Text. The system density is 3.5 nm−3. (TIF) [file pcbi.1008840.s002.tif]

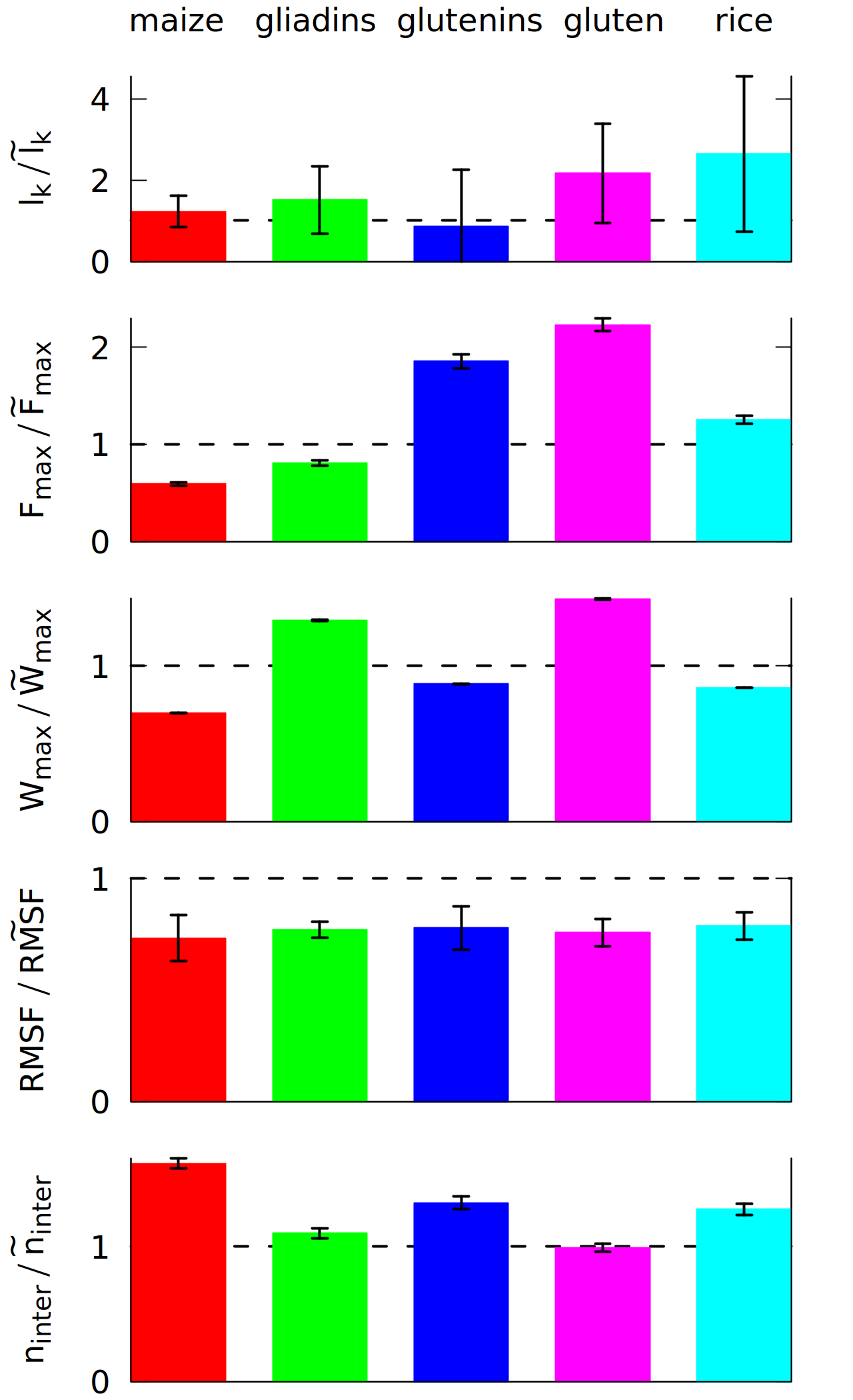

Supplement: S3 Fig — The properties are the average number of entanglements lk, the maximum force Fmax during the elongation in the last simulation stage and the maximum work Wmax required to elongate the system in that stage, the number of inter-chain contacts ninter and RMSF (root mean square fluctuation) averaged over all residues. The ratio of 1 is marked by a broken line. Each color corresponds to a different system, as displayed at the top, and defined in Section 1 of the S1 Text. The system density is 3.5 nm−3. (TIF) [file pcbi.1008840.s003.tif]

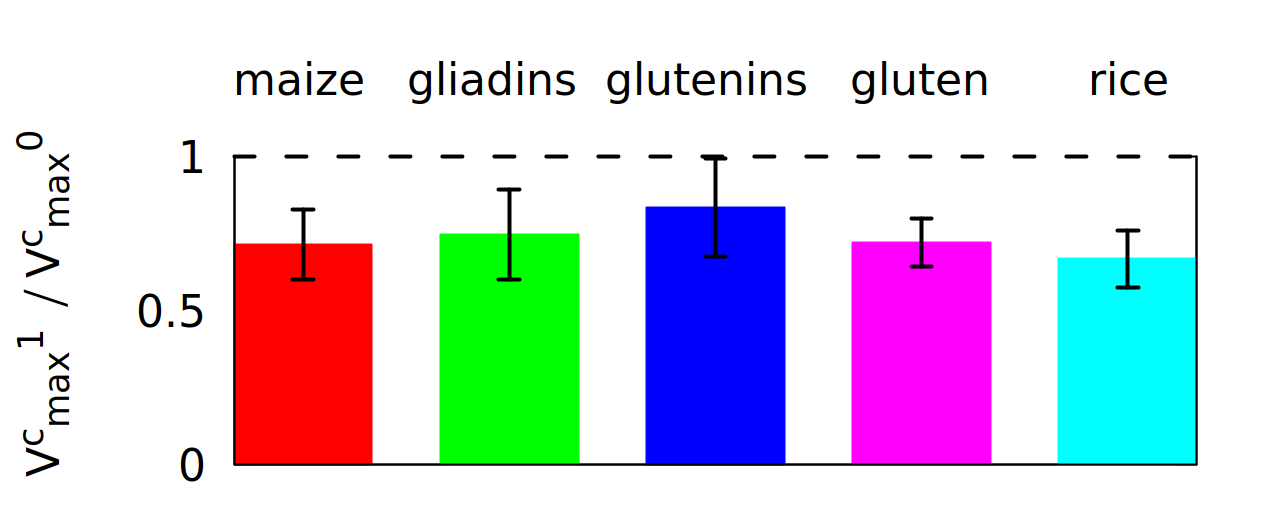

Supplement: S4 Fig — The Vmaxc0 is the average taken over the first half of the elongation process, Vmaxc1 is the average taken over the last half. The ratio of 1 is marked by a broken line. Each color corresponds to a different system, as displayed at the top, and defined in Section 1 of the S1 Text. The system density is 3.5 nm−3. (TIF) [file pcbi.1008840.s004.tif]

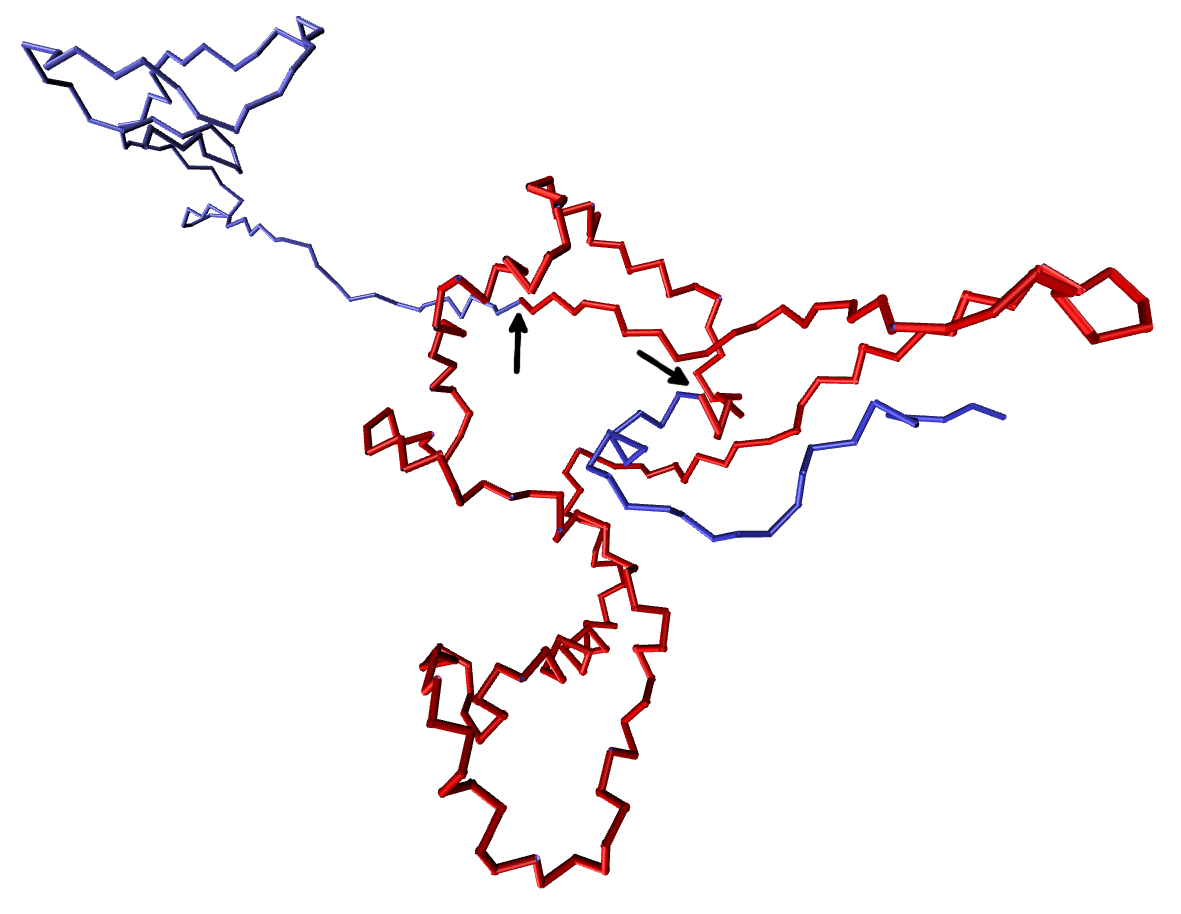

Supplement: S5 Fig — Knotted area is shown in red, knot ends are indicated by black arrows. (TIF) [file pcbi.1008840.s005.tif]

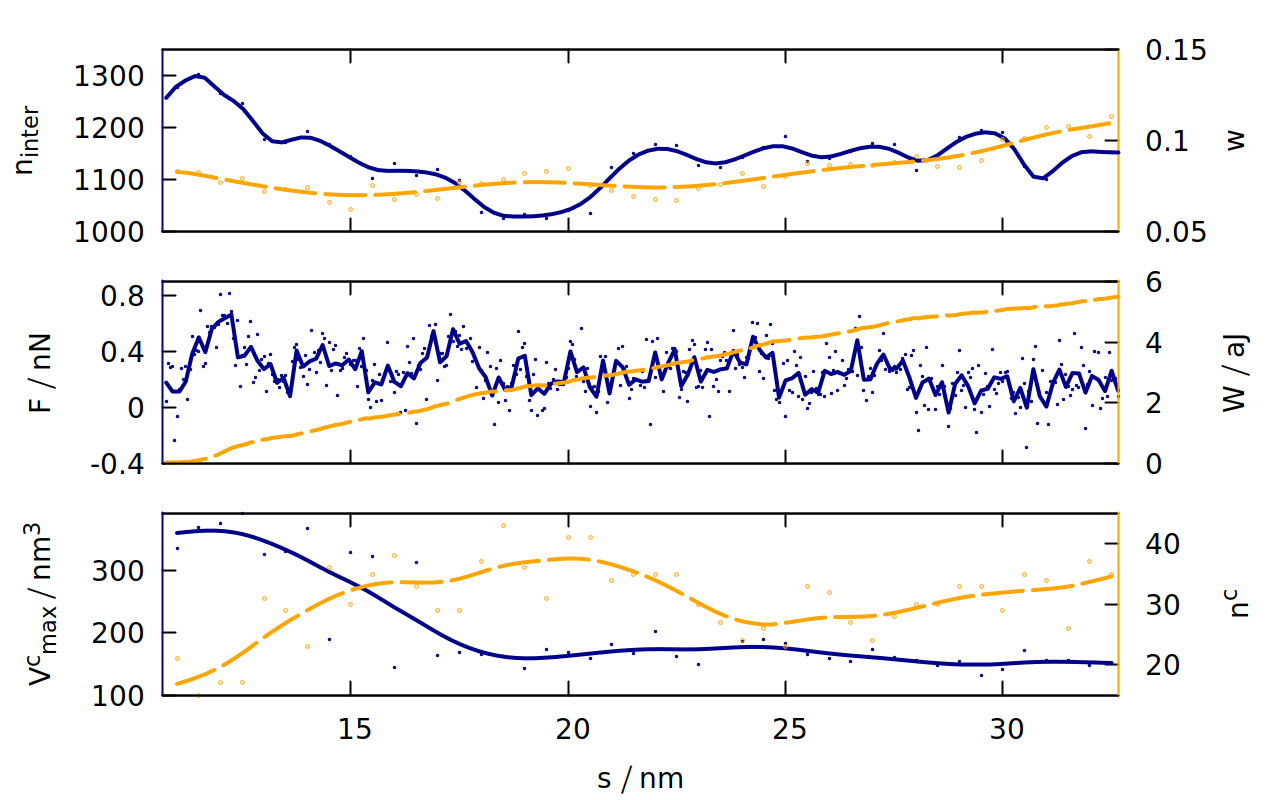

Supplement: S6 Fig — The solid blue (orange) lines correspond to the quantities listed along the left (right) y-axis. Solid (hollow) dots represent raw data from simulation. (TIF) [file pcbi.1008840.s006.tif]
